# The unique value of cardiovascular magnetic resonance in patients with suspected acute coronary syndrome and culprit-free coronary angiograms

**DOI:** 10.1186/s12872-017-0610-6

**Published:** 2017-06-28

**Authors:** Roman Panovský, Júlia Borová, Martin Pleva, Věra Feitová, Petr Novotný, Vladimír Kincl, Tomáš Holeček, Jaroslav Meluzín, Ondřej Sochor, Radka Štěpánová

**Affiliations:** 1 The Department of Cardiovascular Diseases, International Clinical Research Centre, St. Anne’s Faculty Hospital, Brno, Czech Republic; 2 The 1st Department of Internal Medicine/Cardioangiology, International Clinical Research Centre – ICRC, St. Anne’s Hospital, Masaryk University, Pekařská 53, 656 91 Brno, Czech Republic; 3 The Department of Cardiology, Heart Centre, Hospital Podlesi, Trinec, Czech Republic; 4 The Department of Medical Imaging, St. Anne’s Faculty Hospital and Masaryk University, Brno, Czech Republic; 5 International Clinical Research Centre, St. Anne’s University Hospital Brno, Brno, Czech Republic

**Keywords:** Cardiac magnetic resonance, Acute coronary syndrome, Normal coronary angiography

## Abstract

**Background:**

Patients with chest pain, elevated troponin, and unobstructed coronary disease present a clinical dilemma. The purpose of this study was to investigate the incremental diagnostic value of cardiovascular magnetic resonance (CMR) in a cohort of patients with suspected acute coronary syndrome (ACS) and unobstructed coronary arteries.

**Results:**

Data files of patients meeting the inclusion criteria in two cardiology centres were searched and analysed. The inclusion criteria included: 1) thoracic pain suspected with ACS; 2) a significant increase in the high-sensitive Troponin T value; 3) ECG changes; 4) coronary arteries without any significant stenosis; 5) a CMR examination included in the diagnostic process; 6) an uncertain diagnosis before the CMR exam; and 7) the absence of known CMR and contrast media contraindications. Special attention was paid to the benefits of CMR in determining the final diagnosis.

In total, 136 patients who underwent coronary angiography for chest pain were analysed. The most frequent underlying causes were myocarditis (38%) and perimyocarditis (18%), followed by angiographically unrecognised acute myocardial infarction (18%) and Takotsubo cardiomyopathy (15%). The final diagnosis remained unclear in 6% of the patients. The contribution of CMR in determining the final diagnosis determination was crucial in 57% of the patients. In another 35% of the patients, CMR confirmed the suspicion and, only 8% of the CMR examinations did not help at all and had no influence on diagnosis or treatment.

**Conclusion:**

CMR provided a powerful incremental diagnostic value in the cohort of patients with suspected ACS and unobstructed coronary arteries. CMR is highly recommended to be incorporated as an inalienable part of the diagnostic algorithms in these patients.

## Background

Coronary artery disease (CAD) and especially acute coronary syndrome (ACS) are among the leading causes of mortality and morbidity. Detailed guidelines are available for the management and treatment of both acute myocardial infarction (AMI) with and without ST-segment elevations [1, 2]. In addition to several clinical, laboratory and electrocardiographic (ECG) parameters, coronary angiography is one of the basic examinations performed on these patients and in most cases, it provides invaluable information when deciding on the subsequent therapy. However, in several trials, coronary angiography without any visible stenoses/occlusions was found in 1–12% of all patients with ACS [3–9]. In contrast to precise recommendations for the management of proven AMI, there are no guidelines for these culprit-free patients. This means that while the management and treatment of ACS with a clear culprit lesion are nearly identical in all centres, a very different approach is applied to a significant portion of other patients. The methods for clarifying diagnoses and the use of imaging modalities vary in different hospitals. Cardiovascular magnetic resonance (CMR) is a great example of a very useful tool that is used in the vast percentage of cases. While some centres are aware of the great contribution of CMR and its irreplaceability in many cases, other teams use it only rarely.

CMR is a robust non-invasive diagnostic tool in cardiology. It is a precise and highly reproducible technique used to assess ventricular volumes, masses and function. CMR can define cardiac anatomy and structure, quantify myocardial perfusion and measure blood flow. CMR images are acquired without using ionizing radiation or iodinated or radioactive contract agents. Oedema visualization and the late gadolinium enhancement (LGE) technique provide the unique opportunity to assess myocardial tissue in vivo – it enables tissue characterization in ischemic and non-ischemic cardiomyopathies and other cardiac diseases. All these advantages can help to refine the diagnostics of patients with unclear diagnoses [10].

The purpose of this study was to investigate the diagnostic value of CMR in a cohort of patients with suspected ACS and unobstructed coronary arteries.

## Methods

### Patient population and study protocol

The data files of patients meeting the inclusion criteria in two cardiology centres between 2012 and 2016 were searched and analysed. All inclusion criteria had to be fulfilled: 1) thoracic pain suspected with ACS; 2) a significant increase in the high-sensitive Troponin T value over the normal upper limit (value14 ng/l) in at least one value of Troponin T; 3) ECG changes, including at least a 1-mm ST-segment elevation or ST-segment depression or a negative T wave in at least two leads from one coronary artery section; 4) coronary arteries without any significant stenoses (without any atherosclerosis or with a coronary artery stenosis less than 50% in luminal diameter) proven by an admission coronary angiography; 5) a CMR examination included in the diagnostic process; 6) an uncertain diagnosis before the CMR exam; 7) the absence of any known CMR contraindications such as an implanted pacemaker/defibrillator, cochlear implant, ferromagnetic metal parts in the patient’s body or claustrophobia; 8) the absence of contrast media associated contraindications such as significant renal insufficiency; 9) no history or symptoms of former cardiac disease; and 10) the absence of severe cardiac arrhythmias. Special attention was paid to the benefits of CMR in determining the final diagnosis. It was made by comparing the interim diagnosis before CMR (using ECG, laboratory, and coronary angiography results) and the final diagnosis after CMR.

### Cardiac magnetic resonance imaging

CMR studies were indicated after the inclusion criteria were met and were performed according to the standard protocol using 1.5 T scanners (Ingenia and Achieva, Philips Medical Systems, Best, The Netherlands) equipped with 5- and 32-element phased array receiver coils that allow for the use of parallel acquisition techniques in the supine position in breath-hold. Functional imaging was performed using balanced steady state free precession (SSFP, b-TFE) cine sequences with the following parameters: 3.4 ms repetition time (TR), 1.7 ms echo time (TE), 60° flip angle, 1.67 × 1.67 × 8 mm spatial resolution and a 1.5 Sensitivity Encoding – factor. Standard cine imaging including four-chamber, two-chamber, and LVOT (left ventricular outflow track) long axis views, and a short axis (SAX) stack from the cardiac base to the apex in the perpendicular plane to the LV long axis (spatial resolution 1.67 × 1.67 × 1.67 mm, 0 mm gap). Wall motion abnormalities were assessed. LV functional and morphological parameters were calculated from the SA stack using the summation-of-disc method in accordance with the recommendations by the post-processing evaluation of the Society of CMR [11].

Visualization of myocardial oedema was performed using a T2 weighted black-blood image (T2-STIR; TR – 2 R-r intervals, 100 ms TE, 90°flip angle, 1.8 × 1.8x8mm spatial resolution).

LGE images in all long-axis views and the SAX view were acquired 10 min after application of an intravenous bolus of 0.15 mmol/kg of the gadolinium-based contrast agent gadobutrol (Gadovist, Bayer-Schering Pharma, Germany) using a contrast sensitive segmented re-phased turbo field sequence with the slice selective inversion recovery technique (inversion-recovery turbo field echo- IR-TFE). Both 2-dimensional and 3-diamensional data acquisitions were performed in mid-systole with the following parameters: inversion time (TI) 200-300 ms, 3D: TR 4.0, TE 1.3, 15°flip angle, 1.6 × 1.75x5mm spatial resolution; 2D: TR 6.1, TE 3.0, 25° flip angle, 1.6 × 1.9x10mm spatial resolution). The inversion time (TI) for this sequence was optimized on an individual patient basis. LGE was defined as the area of visually identified contrast enhancement that was higher than the mean signal intensity of an adjacent area of the reference myocardium.

The CMR studies were assessed by two experienced interpreters. The information from cine sequences was combined with the T2-weighted imaging and LGE to determine the final diagnosis. All CMR diagnoses - AMI, myocarditis, pericarditis, Takotsubo cardiomyopathy (CMP) and other CMPs were determined according to standard CMR criteria [12]. An increased T2 signal or/and transmural or subendocardial LGE pattern with regional hypokinesia or akinesia in a corresponding coronary artery territory were criteria for unrecognized AMI. For the diagnosis of myocarditis, an increased T2 signal, increased early gadolinium enhancement and non-ischemic (epicardial, mid-wall or patchy) LGE were used as a marker of inflammation. The diagnosis of Takotsubo CMP was made by a combination of transient LV dysfunction (in a typical apical or less frequent mid-ventricular localization) and the absence of LGE.

### Statistical analysis

The baseline characteristics were analysed descriptively and compared between the groups. Standard measures of summary statistics were used to describe the data: relative and absolute frequencies for categorical variables, arithmetic mean supplied with standard deviation for continuous variables.

Most variables did not present a normal distribution (Shapiro-Wilk’s test), therefore non-parametric tests were applied. To compare all groups with continuous parameters, the Kruskal-Wallis test was conducted. To make a detailed mutual comparison of the groups, the Wilcoxon Rank Sum test was performed. The Chi-square test was applied to compare the groups with categorical data.

Results with a *p*-value <0.05 were considered statistically significant.

## Results

The study included a total of 136 patients: 79 (58%) males and 57 (42%) females. The baseline characteristics of the patients are shown in Table 1 - the mean age of the entire population was 48.7 years. One-third of them suffered from arterial hypertension, 8.5% diabetes mellitus, 17% dyslipidaemia, and 28.5% of them were former or current smokers.

**Table 1:** Baseline characteristics

	All population (*n* = 130)	AMI (*n* = 25)	Myocarditis (*n* = 52)	Perimyocarditis (*n* = 24)	Takotsubo CMP (*n* = 21)	Unclear dg (*n* = 8)	*p* among groups
Clinical characteristics							
Age (years)	48.7 ± 18,0	55.9 ± 14.9	41.6 ± 15.8	38.0 ± 17.3	66.2 ± 11.5	58.2 ± 8.9	<0.001*
Female (n)	57 (41.9%)	16 (64.0%)	14 (26.9%)	5 (20.8%)	18 (85.7%)	2 (25.0%)	<0.001*
BMI (kg/m^2^)	27.0 ± 5.1	27.8 ± 6.8	27.6 ± 5.0	24.6 ± 3.6	26.7 ± 4.1	28.1 ± 3.9	0.062
Hypertension (n)	43 (33.1%)	12 (48.0%)	8 (15.4%)	5 (20.8%)	15 (71.4%)	3 (37.5%)	<0.001*
DM (n)	11 (8.5%)	3 (12.0%)	3 (5.8%)	1 (4.2%)	3 (14.3%)	1 (12.5%)	0.640
Dyslipidemia (n)	22 (16.9%)	8 (32,0%)	5 (9.6%)	3 (12.5%)	4 (19%)	2 (25.0%)	0.170
Smoking (n)	37 (28.5%)	10 (40.0%)	13 (25.0%)	9 (37.5%)	4 (19.0%)	1 (12.5%)	0.296
Laboratory							
CK (μkat/l)	15.1 ± 59.0	8.3 ± 6.7	24.1 ± 92.9	13.7 ± 13.1	3.5 ± 2.1	13.9 ± 16.0	0.007
CK-MB (μkat/l)	0.94 ± 0.89	0.93 ± 0.78	0.95 ± 0.85	1.00 ± 1.07	0.63 ± 0.45	1.43 ± 1.46	0.847
Troponin T (μg/l)	0.95 ± 1.07	1.44 ± 1.49	0.77 ± 1.61	1.37 ± 1.42	0.60 ± 0.73	0.34 ± 0.50	0.001*
NT-pro-BNP (ng/l)	1891 ± 4762	1880 ± 3158	1663 ± 6227	895 ± 1095	3999 ± 5107	212 ± 169	0.190
CRP (mg/l)	42.9 ± 50.0	21.0 ± 28.9	46.2 ± 55.6	82.2 ± 76.2	15.3 ± 30.4	21.9 ± 52.1	<0.001*
Echocardiography							
LV EF (%)	53.4 ± 11.2	53.7 ± 10.2	56.1 ± 10.4	53.6 ± 10.3	42.2 ± 10.3	61.3 ± 7.9	<0.001*
WM abnormalities (n)	62 (49.2%)	18 (72.0%)	16 (31.4%)	11 (45.8%)	16 (88.9%)	1 (12.5%)	<0.001*
Coronary angiography							
Coronary AS (n)	43 (35.8%)	11 (44.0%)	12 (25.5%)	3 (15.8%)	12 (57.1%)	5 (62.5%)	0.011*
LV EF (RLVG) (%)	51.3 ± 13.7	56.0 ± 11.0	55.9 ± 13.0	51.6 ± 11.9	37.6 ± 9.4	60.0 ± 9.4	<0.001*

Values are expressed as the mean ± standard deviation or the number of subjects with the percentage in parentheses

*AMI* acute myocardial infarction, *CMP* cardiomyopathy, *dg* diagnosis, *BMI* body mass index, *DM* diabetes mellitus, *smoking* current or prior smoking, *CK* creatine kinase, *CK-MB* creatine kinase-MB isoensyme, *T* Troponin; *NT-pro-BNP* N-terminal fragment of pro-brain natriuretic peptide, *CRP* C-reactive protein, *LV* left ventricle, *EF* ejection fraction, *WM* wall motion, *AS* atherosclerosis, *RLVG* retrograde left ventriculography

* *p* < 0.05 between groups

**Fig. 1:**
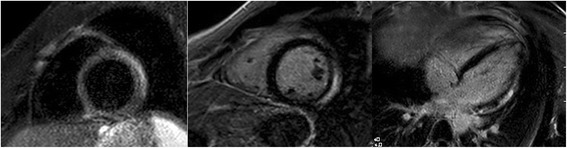
A patient with myocarditis. Detection of myocardial oedema in the anterolateral wall – **a** a short-axis T2- weighted STIR view; **b** a short-axis delayed enhanced view; **c** a four-chamber delayed enhanced view

**Fig. 2:**
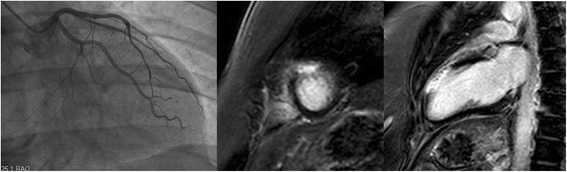
A patient with culprit-free acute myocardial infarction. **a** Left coronary angiography without any stenoses or occlusions; **b** a short-axis delayed enhanced view with a transmural scar in the anterior wall; **c** a two-chamber delayed enhanced view with a transmural scar in the anterior wall

All patients were initially admitted to the coronary unit or the cathlab for chest pain with a suspicion of ACS. The final diagnoses were made after the completion of all examinations, including CMR. CMR was successfully performed in all 136 patients with no adverse events (Table 2). Wall motion abnormalities were found in 62 (47.7%) patients, LV EF was 57.6 ± 12.1%, EDV 130 ± 47 ml, signs of myocardial oedema were diagnosed in 75 (59.1%) patients and myocardial LGE in 94 (72.3%) patients. In 21 (15%) patients, LGE was subendocardial or transmural, while in 73 (54%) patients, LGE was subepicardial, mid-wall or patchy. Twenty-nine patients had pericardial effusion and/or late enhancement of the pericardium. The majority of the patients suffered from inflammatory diseases – myocarditis (*n* = 52, 38%) or perimyocarditis (*n* = 24, 18%) (Fig. 1). No patents had pericarditis alone without any myocardial involvement. Angiographically unrecognised AMI was seen on CMR in 25 patients (18%) (Fig. 2). Takotsubo CMP was diagnosed in 21 patients (15%) as a typical apical ballooning (*n* = 13, 10%) or as the less frequent mid-ventricular form (*n* = 8, 6%). Other CMPs were detected for the first time in 4 patients – 2 (1%) of them had dilated CMP and 2 (1%) had hypertrophic CMP. In 2 patients (1%), the chest pain was concluded to most probably be vertebrogenous, with an unclear reason for Troponin elevation. Despite maximum efforts, the final diagnosis remained unclear in 8 (6%) patients.

**Table 2:** CMR findings

	All population (*n* = 130)	AMI (*n* = 25)	Myocarditis (*n* = 52)	Perimyocarditis (*n* = 24)	Takotsubo CMP (*n* = 21)	Unclear dg (*n* = 8)	*p* among groups
LV EF (%)	57.6 ± 12.1	57.4 ± 13.0	60.1 ± 10.6	55.2 ± 10.8	50.3 ± 12.4	67.9 ± 10.5	<0.001*
EDV (ml)	130 ± 47	112 ± 55	141 ± 41	149 ± 46	103 ± 40	131 ± 34	<0.001*
WM abnormalities (n)	62 (47.7%)	20 (80.0%)	13 (25.0%)	9 (37.5%)	20 (95.2%)	0 (0%)	<0.001*
Myocardial LGE (n)	94 (72.3%)	21 (84.0%)	50 (96.2%)	21 (87.5%)	2 (9.5%)	0 (0%)	<0.001*
Myocardial oedema (n)	75 (59.1%)	15 (65.2%)	37 (71.2%)	19 (79.2%)	4 (19.0%)	0 (0%)	<0.001*
LV hypertrophy (n)	27 (20.8%)	4 (16.0%)	7 (13.5%)	5 (20.8%)	7 (33.3%)	4 (50%)	0.118
Pericardial effusion and/or LGE (n)	29 (22.3%)	3 (12.0%)	3 (5.8%)	23 (95.8%)	0 (0%)	0 (0%)	<0.001*

Values are expressed as the mean ± standard deviation or the number of subjects with the percentage in parentheses

*AMI* acute myocardial infarction, *CMP* cardiomyopathy, *dg* diagnosis, *LV* left ventricle, *EF* ejection fraction, *EDV* end-diastolic volume, *WM* wall motion, *LGE* late gadolinium enhancement

* *p* < 0.05 between groups

For a detailed analysis, 130 patients were included; patients with infrequent diagnoses (dilated CMP, hypertrophic CMP, and vertebrogenous disorders) were excluded from the comprehensive analysis. In line with the expectations, patients with inflammatory diseases were younger than others and had higher values of C-reactive protein. Takotsubo CMP was found more frequently in women. LV EF measured close to admission was lowest in the Takotsubo group, followed by rapid recovery (lower values in earlier vetriculographic and echocardiographic measurements compared to later CMR).

From the whole cohort, 121 (89%) patients had at least one pathology on CMR, while 15 (11%) patients had a completely normal CMR – 8 patients had unclear diagnoses, 2 had vertebral chest pain, 1 had Takotsubo CMP, 1 had histologically proven myocarditis, and 3 had a final diagnosis of more probable small AMI caused by a coronary artery spasm or small distal embolization despite the absence of a visible oedema or a scar on CMR.

All included patients had uncertain diagnoses before the CMR exam, as this was one of the inclusion criteria. On the other hand, all patients were suspected of having some type of pre-CMR diagnosis, but usually there was insufficient data for making such a diagnosis, and it was often poorly defined and chaotic. In 48 (35%) patients, CMR confirmed this previous suspicion and contributed to the diagnostic conclusion. The incremental contribution of CMR for determining the final diagnosis was crucial in 74 (57%) of the patients, where the diagnosis would have remained unclear or incorrect without using CMR - the greatest benefit of CMR was found in patients with unrecognized AMI (76% of them were diagnosed mainly by CMR) and inflammatory diseases (64% of the conclusions were reached only through the use of CMR). Only 11 (8%) CMR examinations did not help at all and had no influence on either the diagnosis or treatment (Table 3).

**Table 3:** CMR contribution for the final diagnosis

	All population (*n* = 130)	AMI (*n* = 25)	Myocarditis (*n* = 52)	Perimyocarditis (*n* = 24)	Takotsubo CMP (*n* = 21)	Unclear dg (*n* = 8)	*p* among groups
Diagnosis made mainly by CMR (n)	74 (56.9%)	19 (76.0%)	31 (59.6%)	18 (75.0%)	6 (28.6%)	0 (0%)	<0.001*
CMR confirmed previous suspicion (n)	45 (34.6%)	4 (16.0%)	20 (38.5%)	6 (25.0%)	15 (74.4%)	0 (0%)	
CMR did not help (n)	11 (8.5%)	2 (8.0%)	1 (1.9%)	0 (0%)	0 (0%)	8 (100%)	

Values are expressed as the number of subjects with the percentage in parentheses

*AMI* acute myocardial infarction, *CMP* cardiomyopathy, *dg* diagnosis

* *p* < 0.05 between groups

## Discussion

Our study, which evaluated the utility of CMR in a cohort of patients with suspected ACS and unobstructed coronary disease, highlights several important findings. First, it demonstrated that CMR has a high incremental diagnostic value - in more than one-half of the patients, the diagnosis would have remained unclear or incorrect without the use of CMR. Secondly, the study confirmed the results from other trials that the vast majority of patients admitted to hospital with a suspicion of ACS suffered from an inflammatory disease, which should have been the main differential diagnosis. Third, our study showed the importance of CMR in cases of AMI in patients in combination with the absence of coronary artery occlusions or stenoses.

Differently than the other trials describing CMR examination in similar cohorts of patients, our work calculated the incremental contribution of CMR for determining the final diagnosis. In more than one-half of the cases (57%), the final diagnosis was either incorrect or uncertain and the benefit of CMR was absolutely essential. In another one-third of the patients, CMR was useful for confirming the diagnoses. These results strengthen the need for using CMR in unclear ACS cases. As in many centres, CMR has not yet been included into the standard approach for these patients and our study aims to encourage making a change in these procedures, because the final diagnosis influences prescriptions for treatment. In literature, CMR diagnosis is reached in 90–93% of all cases [9, 13–15] and CMR leads to a change in therapy in 32% of the patients [16]. Making the correct diagnosis is essential for providing adequate treatment and the appropriate risk-stratification of patients. Although the study samples in the trials were heterogeneous, some studies and meta-analysis described even worse prognoses for these patients (4.7% had a 12-months mortality) compared to patients with AMI [17].

In our study, the majority (56%) of the patients suffered from inflammatory diseases – myocarditis or perimyocarditis, while unrecognised AMI was found in 18% of the patients and Takotsubo CMP was present in 15% of the patients. The distribution of diagnoses differs among studies, mainly due to different inclusion criteria, for instance ECG changes or laboratory markers. Despite the differences in the percentage of diseases, unrecognized AMI and myocarditis are consistently the most frequent causes. In concordance with our study, very similar frequencies of diagnoses have been found by several groups. For instance, in a study by Leurent et al. [5], myocarditis was found in 59.9%, Takotsubo syndrome in 14% and myocardial infarction in 15.8% of the patients. Similarly, Stensaeth et al. [6] described myocarditis or pericarditis in 56% and stress CMP in 10% of the patients, while Assomull et al. [18] found myocarditis in 50%, AMI in 11.6% and CMP in 3.4% of the patients in their trial. Laraudogoitia Zaldumbide et al. [19] discovered myocarditis in 63%, AMI in 15% and Takotsubo CMP in 11% of the patients, and Avegliano et al. [20] diagnosed myocarditis in 60.9%, AMI in 18.7% and stress CMP in 12.5% of their patients. All these trials had very similar inclusion criteria and fewer patients than our study. Some other studies differed greatly, such as a study by Steen et al. [13] in which only 17% of the patients had myocarditis and 38% had AMI. These differences can be explained by their different inclusion criteria, especially by the absence of coronary angiography before CMR. The fact that Mahmoudi et al. [21] performed a CMR examination 2 months after coronary angiography together with missing T2 weighted sequences in the protocol, could probably explain their very low number of myocarditis patients (16%) – it could be assumed that most myocarditis patients were not diagnosed by this late CMR examination. From this comparison, we can highlight the necessity for early inclusion of CMR into the diagnostic algorithm.

CMR is an unbeatable non-invasive method for diagnosing myocarditis. The diagnostic process in myocarditis is based mainly on CMR and endomyocardial biopsy and would be very difficult without using them. Myocarditis is the third leading cause of sudden death and up to 30% of the cases can progress to dilated CMP, so that making the proper diagnosis is crucial for patient management [22, 23]. In our study, 56% of the patients had myocarditis alone or perimyocarditis. Frequently, the first clinical manifestation looks like an AMI, with oppressive chest pain, ECG changes and elevated markers of myocardial damage, so ACS is often the initial diagnosis. CMR provides functional information, but its unique contribution is in the structural information, especially in the detection of regional differences in tissue characteristics. For myocarditis, an increased T2 signal indicating increased water content in an inflamed myocardium, increased early gadolinium enhancement and epicardial, mid-wall, or patchy LGE are typical findings [4, 24]. However, recent data has described a very rapid decrease of CMR oedema markers in just a few weeks with the urgent need for CMR examination at an early stage of the disease [25]. In our study, the mean time interval between the presentation of symptoms and the CMR scan was 7.44 ± 10.03 days, so there is a high probability that all myocarditis patients were captured. The younger age and higher C-reactive protein (CRP) values in myocarditis patients are in concordance with the literature [8].

Culprit-free AMI was the second most common cause in our cohort. The 18% prevalence of these patients is consistent with the published data [8]. Several mechanisms could be proposed to explain these patients, such as coronary spasms, embolisms, atherosclerotic plaque disruptions, or spontaneous recanalization of transitory occlusions. Coronary angiography is a routine examination in patients with ACS. In cases where no visible stenoses/occlusions are found, intravascular ultrasound (IVUS) and/or coronary optical coherence tomography (OCT) can be considered. However, independently of IVUS or OCT results, the diagnosis should be confirmed by other methods. CMR with scar (and/or oedema) visualization is a perfect choice in these situations [26, 27]. A subendocardial or transmural LGE pattern in a corresponding coronary artery section leads to the correct diagnosis with subsequent therapeutic consequences and patient follow-up.

There is no specific CMR pathology for Takotsubo CMP. The diagnosis is usually made by combining transient LV dysfunction and the absence of LGE. Wall motion abnormalities could be apical in typical apical ballooning syndrome, or less frequently as mid-ventricular. Previous studies have shown a prevalence of stress CMP between 11 and 22% [8]; in our cohort, it was 15%.

In a small number of patients (8%), the final diagnosis remained unclear even after using CMR. This number is in keeping with other studies [5]. These patients had lower Troponin T levels compared to other groups and the best LV function among the groups. We could speculate about missing a very small LGE in case of microinfarctions or very mild inflammation due to limited spatial resolution or imaging artefacts. Some cases may be due to an underlying non-cardiac disease. The CMR protocol was not designed to exclude non-cardiac reasons for chest pain and biomarker elevation. Further investigation of these patients after excluding a cardiac origin of their troubles was typically not performed in our centre. There is also the possibility of false positive biochemical results.

### Study limitations

Even though this is not a prospective study, our unit’s policy is to perform CMR in all culprit-free ACS patients. Despite the fact that this is one of the largest cohorts that has been described so far, the relatively small sample size limits the statistical power of our calculations. Another limitation is that the final diagnoses were based on a synopsis of all clinical, ECG, laboratory and imaging findings, including CMR. Individual examinations differed slightly according to each patient’s medical history and interim results. Nevertheless, this bias reflects an actual patient workflow.

Especially for patients with AMI, IVUS and/or OCT could help in the understanding of the aetiology, but these procedures were not routinely performed. Furthermore, MR stress first-pass perfusion and cardiac mapping sequences and extracellular volume were not included in the standard CMR protocol. They would certainly provide additional useful information.

## Conclusion

Our study demonstrated that CMR had a powerful incremental diagnostic value in a cohort of patients with ACS and unobstructed coronary arteries. CMR is strongly recommended to be included as an inalienable part of diagnostic algorithms in these patients, because making the correct final diagnosis has direct implications on patient management.

## Abbreviations

ACS: Acute coronary syndrome
AMI: Acute myocardial infarction
AS: Atherosclerosis
BMI: Body mass index
CAD: Coronary artery disease
CK: Creatine kinase
CK-MB: Creatine kinase-MB isoenzyme
CMP: Cardiomyopathy
CMR: Cardiovascular magnetic resonance
CRP: C-reactive protein
DM: Diabetes mellitus
ECG: Electrocardiography
EDV: End-diastolic volume
EF: Ejection fraction
EGEr: Early gadolinium enhancement ratio
IR-TFE: Inversion-recovery turbo field echo
IVUS: Intravascular ultrasound
LGE: Late gadolinium enhancement
LV: Left ventricle
LVOT: Left ventricular outflow track
NT-pro-BNP: N-terminal fragment of pro-brain natriuretic peptide
OCT: Optical coherence tomography
RLVG: Retrograde left ventriculography
SAX: Short axis
SSFP: Steady state free precession
TE: Echo time
TI: Inversion time
TR: Repetition time
WM: Wall motion
